# Variation in Global Policy Responses to COVID-19: A Bidirectional Analysis

**DOI:** 10.3390/ijerph20054252

**Published:** 2023-02-27

**Authors:** Caixia Wang, Huijie Li

**Affiliations:** 1 Qu Qiubai School of Government, Changzhou University, Changzhou 213159, China; 2 Institute of Public Agency Administration, Changzhou University, Changzhou 213159, China; 3 School of Public Administration, Jilin University, Changchun 130012, China

**Keywords:** COVID-19, policy response, health capacity, vaccination coverage, deaths

## Abstract

Against the unprecedented outbreaks of the COVID-19 variants, countries have introduced restrictive measures with discretion, ranging from lifting the closure thoroughly to implementing stringent policies, but all together guarding the global public health. Under the changing circumstances, we firstly apply the panel data vector autoregression (PVAR) model, using a sample of 176 countries/territories from 15 June 2021 to 15 April 2022, to estimate the potential associations among the policy responses, the progression of COVID-19 in deaths and vaccination, and medical resources possessed. Furthermore, we use the random effect method and the fixed effect speculation, to examine the determinants of policy variances across regions and over time. Our work has four main findings. Firstly, it showed the existence of a bidirectional relationship between the policy stringency and variables of interest including new daily deaths, the fully vaccinated percentage and health capacity. Secondly, conditional on the availability of vaccines, the sensitivity of policy responses to the death numbers tends to decline. Thirdly, the role of health capacity matters in coexisting with the virus mutation. Fourthly, regarding the variance in policy responses over time, the impact of new deaths tends to be seasonal. As to geographical differences in policy responses, we present the analysis for Asia, Europe, and Africa, and they show different levels of dependencies on the determinants. These findings suggest that bidirectional correlations exist in the complex context of wrestling with the COVID-19, as government interventions exert influence on the virus spread, the policy responses also progress alongside multiple factors evolving in the pandemic. This study will help policymakers, practitioners, and academia to formulate a comprehensive understanding of the interactions between policy responses and the contextualized implementation factors.

## 1. Introduction

Adapting policy responses to the evolving pandemic status is tricky for most countries. It has been three years since the World Health Organization (WHO) declared COVID-19 a global pandemic. To contain the emerging waves of the COVID-19, government interventions have become common practice [1,2]. Extensive research has evidenced their effectiveness on health outcomes [3,4,5,6]. Choi et al. (2022) demonstrate that fewer cases and deaths correlate with the global restrictive movement [7]. Non-pharmaceutical interventions (NPIs) have been identified to minimize morbidity and mortality prior to the introduction of vaccines, and it is suggested that NPIs can be lifted as COVID-19 vaccination campaigns progress [8]. However, uncertainties lie in the growing variants, deaths and social/economic disruptions [9]. Compared with the original strain of the virus, the mutations of COVID-19 tend to be more vaccine-resistant, and Delta causes more severe diseases with increased probability [10]. People have double the hospitalization risk with Delta than they did with Alpha [11]. Owing to Omicron’s higher transmissibility, the lower hospitalization rate can still lead to a high number of hospitalizations overall [12]. Along with the debate on loosening the strictness of policy responses, WHO has viewed the COVID-19 pandemic, despite its present status as a public health emergency of international concern (PHEIC), as probably being at a transition [13].

In response to the COVID-19 variants, counties have implemented diverging policy measures. The vaccine penetration stands as the basis for easing policy responses. Reaching the herd-immunity threshold depends on the combination of multiple factors, covering vaccine hesitancy and willingness for uptake, the emergence of new variants and the delayed arrival of vaccinations for children [14]. When surges spread in the most vaccinated states, experts urge people to get booster shots [15]. Before achieving a desirable vaccination rate, stringent policies can target suppressing the case numbers of COVID-19, but such a strategy has limitations in its effectiveness when confronted with more infectious variants, such as the Omicron variant. As expected, the growth of vaccination rates combines with the continuing function of policy response to allow the reduction of restrictions [16]. Similarly, in the case of Europe, broad vaccination coverage helps to cut down the peaking of death rates, which safeguards some countries able to ease the government interventions; whereas, some other countries may still struggle at the earlier stage of the COVID-19 pandemic [17]. The Delta and Omicron variants are more transmissible, even among vaccinated people, and resuming normal life can also be disrupted by prevalent infections and clustered outbreaks.

Owing to the Oxford COVID-19 Government Response Tracker, the world has witnessed comparable variations in the interventions of countries [2]. A tale of two ends in the wrestling shows that, when some countries still fight with caution, the remaining ones choose to loosen overall restriction measures, even though case counts reached recorded highs during the Omicron surge [18]. The existing literature has substantially evaluated the functions of particular policies from individual aspects or country views, by focusing on the years before Omicron or considering small cohorts of countries [19,20,21]. However, there are insufficient research concerns about the global application status from a more macro perspective, above the country level, so that governments can learn from each other even under their own circumstances. As we understand the respective efforts of countries in the global pandemic fight, then the world can be better prepared for future infectious disease threats. On the one hand, countries have implemented diverging interventions to respond to the high uncertainty generated from the COVID-19. The rising availability of effective vaccines and high immunity rates promise the lifting of restrictions [8]. For example, at the beginning of 2022, Australia showed a reduction in the stringency of policy responses after reaching high vaccination rates, as Omicron was revealed to be less fatal and cause lower hospitalization rates [16]. On the other hand, the pandemic situation not only exerts one-way influence on the policies to be implemented, but the policy responses co-evolve with those factors that affect its progression, such as India [22]. Policy implementation correlates with health outcomes and pushes the global pandemic response forward. With these doubts, we selected the waves of Delta and Omicron to speculate about the correlations between the variation in policy reactions to COVID mutations and health outcomes.

Existing literature normally performed one direction analysis of the effectiveness of worldwide government interventions [3,4,5], or vaccination impact on policy stringency [8,23], but little discussion has been set in the dynamic co-evolution context of vaccination rates, infection status and policy stringency. The role of medical-care supplies in influencing policy adjustments deserves further exploration. The success of mitigating case counts and deaths has partly relied on the conditions and accessible resources of countries, in particular, population density and health service coverage. Purnomo et al. (2022) identified the varying extent of policy responses to the COVID-19 pandemic with an emphasis on the health capacity of countries [24]. Broad policy interventions were found to be correlated with a short-term reduction in death rates [25]. Hamid et al. (2022) recognized contingency interventions at the optimal time in controlling the COVID-19, and found that relatively earlier termination of NPIs is inclined to trigger new waves of infections [26]. To measure the developments of capabilities to address infectious disease threats, GHS (global health security) indicators have been found to associate strongly with the cumulative COVID-19 deaths [27].

This study makes contributions primarily from two aspects. Firstly, in comparison with the work of Dergiades et al. (2022), who assessed the impact of non-pharmaceutical interventions on deaths in the first wave of the COVID-19 [5], our study focuses on the waves of Delta and Omicron within a broader time frame of 15 June 2021 to 15 April 2022. This investigation touched upon the whole dynamic context rather than emphasizing the unidirectional impact of constraining policies and the pandemic progression. It may be more appropriate to explore the association between policy responses and daily death counts in the dynamic context, taking the feature changes of the virus into consideration. Secondly, with the resurgence of different waves of COVID-19 outbreaks, the intervention in itself may impact later adjustments as well as health outcomes; moreover, deaths could also exert influence on the policy responses. Additionally, we also explored the heterogeneity of policy responses across regions and over time.

## 2. Model, Data and Method

As suggested above, to investigate the dynamics of policy responses during the evolution of COVID-19, we build a dataset by matching the Stringency Index dataset, global COVID-19 time-series data, global vaccination data, population data from the World Bank, and global health security index. There are 176 countries and territories in the sample, as presented in Table S1 of the Supplementary Materials, covering the time period between 15 June 2021 and 15 April 2022. The analysis contains the variables presented in Table 1. Descriptions of the variables can be found in Table S2, and Table S3 for the information of stringency index components, and Table S4 verifies the cross-sectional dependence among variables.

With respect to interest variables, we proxied policy responses using the Stringency Index from the Oxford Coronavirus Government Response Tracker (OxCGRT) [2]. The stringency index measures the level of government non-pharmaceutical interventions with a range of 0 to 100 [28]. Regarding global COVID-19 time-series data, we used new daily deaths in the models and included new daily confirmed cases in the robustness check section; both of these referred to the counts, including confirmed and probable (where reported) cases, and they represent the evolution of COVID-19 diffusion at the cross-sectional level over time [29]. To measure the variations of vaccination distribution in countries, we computed a variable of fully vaccinated percentage by combining the global vaccination data and the population data of World Bank [30]. In particular, we used the variable of ‘people_fully_vaccinated’ and ‘people_vaccinated’ in the whole population of countries/territories to obtain fully vaccinated percentage and vaccinated percentage, denoted by ‘vacci_fpc’ and ‘vacci_pc’, respectively [31]. Such a computed variable contains the updated population data, which followed the insight from our earlier study that emphasized the influence of population density in fighting against the first wave of COVID-19 [32]. The variable of health capacity refers to that of clinics, hospitals and community care centers; it is a sub-indicator of the health system in the Global Health Security (GHS) Index 2021. According to the index criteria, a score from 0 to 100 represents the level of health capacity, 0 represents the lowest, and 100 signifies the highest [33].

Considering our concern is the possible relationships among multiple variables, in the section of bidirectional analysis, we apply the time-series vector autoregression (PVAR) estimation by running regression analyses with STATA 15. The PVAR approach is of joint advantages from the traditional VAR approach and the panel data approach, as it treats all variables as endogenous without strict assumption about the exogenous issue, and allows for unobserved individual heterogeneity [34,35]. These advantages fit well with the COVID-19 variants and the dynamics and diversity of policy responses. There are four PVAR models. Model 1 presents the context where the relationship between policy response and new daily deaths was taken into consideration, without vaccine applications. Model 2 adds the impact of vaccination on lifting the policy response, which underpins the logic of the countries that have claimed the ending of COVID-19. Along with the policy adjustment, Model 3 aims to point out the effect of the health system exposed upon the policy space in public health crisis, while countries differ in possessing medical resources. Model 4 is a combination of vaccination factor and health capacity. Differentiating from the PVAR models, Model 5 emphasizes the policy variance across regions using the random effect method, and Model 6 undertakes the fixed effect speculation to examine the variance over time. Relevant equations in these models can be found in the Table S5 in the Supplementary Materials.

To prove that the dataset satisfies the assumptions of the PVAR models, we tested each variable for stationarity using unit root tests. Given the existence of missing values varying from the interest variables, although the dataset indicated a strongly balanced structure, the actual distributions of covariates presented another picture. On basis of such an internal property of the dataset, we use the Im-Pesaran-Shin (IPS) test and Fisher test [36]. These tests also embed the lag orders in the four PVAR models respectively. The optimal lag order varies from models, which should be determined through the AIC, BIC, and HQIC method [37]. In this study, we basically follow the model selection criteria by Andrews and Lu (2001) [38]. Apart from this, we choose the simpler equation structure to the standard of MBIC when confronted with a lower value of MBIC and a higher value of MAIC and HQIC, based on the theoretical argument that COVID-19 variants mutate faster with invisible symptoms. As is shown in the Table S7 of the Supplementary Materials, the optimal lag order of the stringency index and new deaths in Model 1 is 1, the optimal lag order of stringency, new deaths, and fully vaccinated percentage in Model 2 is 2, the optimal lag order of stringency, new deaths, and health capacity in Model 3 is 3, and the optimal lag order of stringency, new deaths, fully vaccinated percentage and health capacity in Model 4 is 2. In terms of the unit root tests, the null hypothesis is that all individuals would follow a unit root process, while the alternative one allowed some of the individuals to have a unit root process. According to the results reported in the Table S6 of the Supplementary Materials, all the results of the Fisher tests reject the null hypothesis at a P value of 0.01, as is the same with some of the IPS results, while the absence of partial results was attributed to the low compatibility of data quality and test application. Panel VAR models 1–4 were tested to be stable, which is shown in Tables S8–S10 of the Supplementary Materials.

Model 5 and Model 6 speculate the variance of policy in stringency across regions and over time. Following the previous application of PVAR in Model 4, we have lagged the variable of new daily deaths and fully vaccinated percentage by the order of two days, considering that it takes time for policymakers to have a comprehensive knowledge of the infection status. The data for health capacity has been collected by year, thus we keep its original value. In the regional analysis, we made a comparison between Asia, Europe, and Africa. As policy coordination in responding to COVID-19 is weak, we use the random effect model with countries grouped in Model 5. Table 6 examines the variance in policy responses based on the monthly analysis by applying the fixed effect model.

## 3. Results

In this section, the main results are composed of the bidirectional analysis with Model 1, Model 2, Model 3 and Model 4, and the comparative analysis using Model 5 and Model 6.

### 3.1. Bidirectional Analysis

Table 1 reports the descriptive statistics of four variables related to COVID-19. As is shown by the stringency index, countries differ in the strictness of responses towards COVID-19 variants. The number of daily deaths has a right-skewed distributional structure, and the distribution of the fully vaccinated percentage is left-skewed. For the health capacity, it shows a large gap between countries with robust health systems and those with quite limited health resources.

Table 2 and Table 3 show the outcomes of the PVAR models. They indicate that as the policy stringency increases, other variables respond too. So as for the new daily deaths, fully vaccinated percentage, and health capacity. The results point to the existence of a bidirectional relationship between the policy stringency and new daily deaths, the fully vaccinated percentage and health capacity in the model. Most of the correlations tend to be weaker over time. Table 2 reports the composite results of Model 1, Model 2 and Model 3. Model 1 shows that, new deaths per day have positively contributed to the level of the stringency index. As for the effect of the stringency index on new daily deaths, the coefficient is significantly negative. This result demonstrates the predicted impacts of policy responses on the death toll of COVID-19. In Model 2 which added the fully vaccinated percentage, the effect of policy stringency on mitigating the virus spread is greatly heightened. The increase of the fully vaccinated percentage brings about a decrease in new deaths, which is in line with research that has confirmed the positive effect of vaccination [39]. Conditional on the availability of vaccines, the sensitivity of policy responses to the death numbers tends to decline. For Model 3, the effect of the stringency index on new daily deaths remains negative. As for the impact of new deaths on the stringency index, a strengthening trend tends to exist over time. The stringency index and health capacity are found to be positively correlated. Table 3 reports the results of Model 4. The health capacity and the fully vaccinated percentage is indicated to be positively correlated. A new finding is that, the strengthened health capacity could contribute to decreasing the number of new daily deaths, the mitigation effect of which is higher than the vaccination.

### 3.2. Comparative Analysis

#### 3.2.1. Regional Analysis

The pace of the COVID-19 pandemic is different across regions, as well as the policy responses, according to their respective circumstances. Table 4 shows that, in comparison with Europe and Africa, Asia is the region which has the largest differences in the stringency index (SD = 17.180) among countries, with the maximum value of over 90, while Europe has the lowest standard deviation with a higher minimum value than Asia and Africa. In Table 4, we can see the maximum number of new deaths per day in Asia was recorded at 4100, while the numbers were 1222 and 953 in Europe and Africa, respectively. This argument is highly dependent on the reported deaths data, without considering reporting bias and excess mortality. Viewing the fully vaccinated percentage and health capacity, Africa is the region that has the least medical assets when dealing with the COVID-19.

What factors affected the geographical variance of policy responses? Table 5 gives the answers for Asia, Europe and Africa. Column (1) reports the results from Asia. The lagged fully vaccinated percentage is found to be the most influential variable with the coefficient of −0.171 on the stringency index. New daily deaths is found to be positively correlated with the stringency index. The correlation between health capacity and stringency index is not significant, as well as the interaction effect between new deaths and fully vaccinated percentage on policy stringency. Yet health capacity is found to exert indirect influence by its interaction with deaths and vaccination. In Column (2) of Table 5, for Europe, only the variable of fully vaccinated percentage has a statistically negative effect on the stringency index, but the coefficient is −0.148, slightly less than that of Asia. In Column (3), health capacity stands out as the most influential explanatory variable for Africa, with a coefficient of 0.527. But it is contrary to common sense that countries with better health capacity normally have less pressure in making more restrictive policy responses to the COVID-19 pandemic. This may indicate that the policy attention to the pandemic matched the level of medical resources able to be retrieved for developing countries. Taken together, Africa is the region where policy responses corresponded most strongly to new deaths, fully vaccinated percentage, and health capacity.

#### 3.2.2. Monthly Analysis

The distribution of policy stringency fluctuated during the research period. As shown in Table 6, the zero value only existed in July 2021. For most of the time, the scale of policy stringency stays at a very low level of 2.78, aside from peaking in November 2021 and December 2021. For the maximum value, there were more waves for the fluctuation, but since March 2022 it showed a sign of loosening. The mean value illustrates a clearer tendency for the decreasing scale of policy stringency.

How will policy responses to the COVID-19 pandemic vary over months? Table 6 reports the results between June 2021 and April 2022, and Table 7 for the determinants of policy variances over time. The effect of deaths on policy stringency tends to be seasonal, in particular more apparent in February and in September. As policy interventions may stay for several months, this explains why its stringency was insensitive to deaths between October 2021 and December 2021. During this period, the health capacity mattered more. Since March 2022 as a transition point in the policy stringency, even with the increasing number of new daily deaths, the policy responses to the pandemic was lifted. Fully vaccinated percentage kept its role in decreasing the scale of stringency. For the most of the time, both the fully vaccinated percentage and health capacity are found to be negatively associated with the policy stringency. The coefficient of the fully vaccinated percentage is greater than that of health capacity on the policy responses, which indicates that the health capacity received less attention from policy-makers for health emergencies. In addition, all the interaction effects are marginal.

### 3.3. Robustness Check

For the bidirectional analysis, we adopt two strategies to check the robustness of estimates by using alternative independent variables in the PVAR models (Robustness Check 1 in the Supplementary Materials) and the alternative method with the components of the former dependent variables (Robustness Check 2 in the Supplementary Materials). The results of the robust models are basically in line with the findings above, coinciding with the specification of Agyapon-Ntra and McSharry (2022) [4] that the impacts of diverse policies in response to COVID-19 differ with the evolution of the virus. For details, please see Tables S16–S30. To check the robustness of the results generated from the comparative analysis, we also ran regressions with the sub-datasets of North America and South America, respectively. Similarly, the impact of daily new deaths on the strictness of policy in these two regions are convincingly positive, and detailed results can be found in the Robustness Check 3 of the Supplementary Materials, which are Tables S31 and S32.

## 4. Conclusions and Discussion

To examine the complex associations among variables concerning COVID-19 containment, we conducted a layered research design by analyzing four PVAR models. In the dynamic environment of constraining policies and the pandemic progression, policy stringency was found to be correlated to the new daily deaths, vaccination coverage, and the health capacity. During the period between June 2021 and February 2022, the effect of deaths on policy stringency tends to be seasonal, in particular more apparently at the ending month of winter (February) and the starting month of autumn (September). March 2022 was identified as a transition point in the policy stringency, even with the increasing number of new daily deaths, the policy responses to the pandemic were lifted. As to the geographical differences in policy responses, we presented an analysis for Asia, Europe, and Africa, and they showed different levels of dependency on the determinants. For example, Europe is solely influenced by the vaccination coverage shown in Model 5. Our research is in line with the finding that policy responses vary by vaccination status, but the availability of vaccines may not be the single decisive factor in easing policy responses to COVID-19, for regions such as Asia and Africa (see Table 5). With a large population and limited per capita medical resources, even when reaching a high vaccination rate, the adjustment of policy responses still requires discretion in the face of the trends toward ending COVID-19 restrictions.

The argument that adopting policy responses reduces deaths coincides with the work of Hale et al (2021), in which Hale and his colleagues obtained daily data from 1 January 2020, to 11th March 2021, covering different waves in specific countries and considering demographic, health system, and economic characteristics [6]. Whereas, our paper focuses on the waves of Delta and Omicron. This article made the role of full vaccination stand out in relieving the non-pharmaceutical restrictions, but the influence of vaccine supply constraints and the distribution among different age groups [40] should deserve more attention. With the development of virus mutations, the efficacy also evolved [41,42]. Nevertheless, the proactive way of keeping away from COVID death peaks relieved the pressure of complying with stringent policies in emergency periods.

Moreover, we provide new evidence for the correlation between the health capacity and vaccination, between the health capacity and policy stringency. The health capacity and the fully vaccinated percentage is indicated to be positively correlated. The strengthened health capacity could contribute to decreasing the number of new daily deaths, the mitigation effect of which can be higher than the vaccination penetration. Due to data limitation, diagnostic testing policies and contact tracing were not taken into account in the analysis. The regional analysis suggests that policy adjustments to the pandemic can be influenced by the health capacity, while it may reflect different levels of attention from policymakers to the pandemic to match the medical resources required. For example, health capacity stood out as the most influential explanatory variable in Africa, and its effect on policy stringency wass positive, which is contrary to common sense that countries with better health capacity normally have less pressure in making more restrictive policy responses to the pandemic. In the long run, it suggests that more policy emphasis should be given to the systematic improvement of health capacity rather than simply on the vaccination coverage.

To sum up, this study has enriched the established theoretical expositions on the impact evaluation of policy responses for COVID-19 evolution, which casts new light on coordinating global heterogeneous interference at the policy implementation level.

This research has the following limitations. One limitation lies in missing values in the dataset. In particular, the vaccination data observations had the least match to the remaining variables, and this was far from a desirable global dataset that takes the variety of vaccine accessibility, dose times of uptake, and vaccination acceptance into account. This may have affected the presented results of Model 2 and Model 4. For this reason, we chose not to add more variables such as economic expenditures. Another limitation came from the application of the statistical software. PVAR in STATA constrained explorations with a longer time-span. It is widely acknowledged that there is a 14-day window in the virus spreading; however, the technical setting allows for the selection of only one suitable lag order as the default, which may bring unknown time effects in the whole computation process. Lastly, as the proxy of policy responses lacked alternative variables, we could only use the stringency index and its components, including C1, C2, C3, C4, C5, C6, C7, C8 and H1. There may have been gaps between the level of policy stringency and the intensity of enforcement measures, which relates to public behavioral variations over time, such as ‘pandemic fatigue’ [43]. Thus, the efficacy of policy responses to COVID-19 variants may be associated with factors that stand beyond the field of policymaking. In relation to the doubt that policy responses vary during the implementation duration, controlling measures like restrictions on international travel may experience little variance; whereas, the magnitude of social distancing can change in a shorter time. Given that this study proxied policy responses with the stringency index, which was a composite of relevant policies, rather than a single specific measure, this worry did not create a problematic disturbance to the research. Our analysis did not include the effect of masking, which may erode the quality of the discussion for policy responses related to reducing deaths somehow, as wearing masks is quite an effective non-pharmaceutical intervention [4]. In addition, our analysis is based on reported deaths data, which could be biased without considering excess mortality, and the latter varies among counties and regions [44].

Future studies are encouraged to follow the avenue of this research. Considering that local heterogeneity in the exposure of contagion risk hindered the efficacy of regional non-pharmaceutical interventions in Italy [45], this triggers questions about how to include local factors in the effectiveness analysis of policy responses. The magnitude of policy responses differs by the category of political institutions [46]. Should future research be combined with institutional contexts, the global picture for combating the pandemic could be more vivid and convincing. As globally consented, the initial objective of response policies aims to flatten the curve by reducing the spread pattern of the virus, in case an uncontrollable number of people were confirmed to be infected at the same time [47]. But the utopia seems to be broken by unstoppable evolving COVID variants, e.g., BA.5 is faced with undercount partly due to unreported at-home COVID-19 tests [48]. The number of deaths could be undervalued [49]. Now that the end of the COVID-19 pandemic seems to be drawing closer, when can we step into the stable endemic stage? Furthermore, are we fully prepared for other high-threat infectious hazards in the future?

## Figures and Tables

**Table 1:** Descriptive analysis of the variables.

Variables	N	Mean	Standard Deviation	Minimum	Maximum
Stringency Index	52,226	47.063	17.973	0	97.220
New Deaths per day	52,316	44.341	177.395	0	4188
Fully Vaccinated Percentage	24,303	45.703	26.014	0.001	97.398
Health Capacity	51,850	30.966	20.277	0.500	78.900

**Table 2:** PVAR Results of Model 1, Model 2 and Model 3.

Model	Independent Variables	Dependent Variables			
		Stringency	New_Deaths	vacci_fpc	helt_cp
Model 1	stringency (−1)	0.087 *** (0.012)	−2.475 *** (0.114)		
	new_deaths (−1)	0.001 *** (0.000)	0.026 *** (0.005)		
Model 2	stringency (−1)	0.048 (0.042)	−22.603 *** (1.658)	0.094 (0.064)	
	stringency (−2)	−0.176 *** (0.036)	−22.538 *** (1.649)	0.925 *** (0.056)	
	new_deaths (−1)	−0.003 *** (0.001)	−0.042 (0.037)	0.006 *** (0.001)	
	new_deaths (−2)	0.000 (0.001)	0.124 *** (0.038)	0.004 *** (0.001)	
	vacci_fpc (−1)	0.123 *** (0.018)	−6.883 *** (0.933)	0.239 *** (0.029)	
	vacci_fpc (−2)	0.086 *** (0.014)	−4.644 *** (0.586)	0.173 *** (0.023)	
Model 3	stringency (−1)	−0.063 *** (0.008)	−0.416 *** (0.096)		0.403 *** (0.010)
	stringency (−2)	−0.126 *** (0.008)	−0.415 *** (0.119)		0.200 *** (0.010)
	stringency (−3)	0.004 (0.008)	0.025 (0.135)		0.350 *** (0.012)
	new_deaths (−1)	0.001 *** (0.000)	0.033 *** (0.006)		0.003 *** (0.000)
	new_deaths (−2)	0.003 *** (0.001)	0.050 *** (0.005)		−0.001 * (0.001)
	new_deaths (−3)	0.007 *** (0.000)	0.079 *** (0.004)		−0.007 *** (0.000)
	helt_cp (−1)	0.099 *** (0.005)	−0.858 *** (0.035)		0.024 *** (0.006)
	helt_cp (−2)	0.198 *** (0.005)	−0.430 *** (0.035)		0.128 *** (0.007)
	helt_cp (−3)	0.047 *** (0.005)	−1.155 *** (0.043)		0.056 *** (0.006)

Notes: In Model 1, Model 2, and Model 3, there are 48021 observations with 305 panels, 4149 observations with 300 panels and 39963 observations with 305 panels, respectively. Standard errors are in parentheses, *** *p* < 0.01, * *p* < 0.1.

**Table 3:** PVAR results of Model 4.

Model	Independent Variables	Dependent Variables			
		Stringency	new_deaths	vacci_fpc	helt_cp
Model 4	stringency (−1)	0.158 *** (0.035)	−16.222 *** (1.356)	0.042 (0.056)	0.137 *** (0.046)
	stringency (−2)	−0.095 *** (0.030)	−14.679 *** (1.411)	0.876 *** (0.045)	0.244 *** (0.043)
	new_deaths (−1)	0.001 (0.001)	−0.117 *** (0.039)	0.010 *** (0.001)	0.012 *** (0.001)
	new_deaths (−2)	−0.002 ** (0.001)	0.189 *** (0.032)	0.002 (0.001)	0.015 *** (0.002)
	vacci_fpc (−1)	0.190 *** (0.018)	−2.585 *** (0.904)	0.233 *** (0.030)	0.319 *** (0.021)
	vacci_fpc (−2)	−0.001 (0.019)	−0.397 (0.643)	0.089 *** (0.029)	0.118 *** (0.026)
	helt_cp (−1)	0.011 (0.017)	−9.027 *** (0.489)	0.108 *** (0.029)	−0.103 *** (0.027)
	helt_cp (−2)	0.266 *** (0.030)	−10.529 *** (1.124)	0.244 *** (0.047)	0.078 ** (0.039)

Notes: There are 4098 observations with 300 panels; Instrument: 1(1/2). (stringency new_deaths vacci_fpc helt_cp); Standard errors are in parentheses, *** *p* < 0.01, ** *p* < 0.05.

**Table 4:** Descriptive analysis of the variables by region.

Regions	Variables	N	Mean	Standard Deviation	Minimum	Maximum
Asia	Stringency Index	12,268	53.865	17.180	2.78	93.52
	New Deaths per day	12,499	52.279	154.306	0	4100
	Fully Vaccinated Percentage	6771	45.788	26.153	0.029	97.398
	Health Capacity	12,200	35.695	17.606	1.8	75.6
Europe	Stringency Index	12,581	42.862	14.842	8.33	84.26
	New Deaths per day	12,471	54.628	147.477	0	1222
	Fully Vaccinated Percentage	7947	55.791	19.770	0.653	91.434
	Health Capacity	12,200	48.045	15.038	13.1	78.9
Africa	Stringency Index	15,088	41.365	17.049	2.78	87.04
	New Deaths per day	15,548	7.525	33.455	0	953
	Fully Vaccinated Percentage	2833	13.547	16.218	0.001	81.405
	Health Capacity	15,555	14.180	11.292	0.5	45.2

**Table 5:** Variance in policy responses across regions (Model 5).

Independent Variables	Dependent Variable: Stringency		
	(1) Asia	(2) Europe	(3) Africa
new_deaths (−2) (I)	0.030 *** (0.007)	0.004 (0.009)	0.091 *** (0.011)
vacci_fpc (−2) (II)	−0.171 *** (0.008)	−0.148 *** (0.012)	−0.451 *** (0.023)
helt_cp (III)	0.122 (0.107)	−0.042 (0.103)	0.527 *** (0.164)
Interaction (I × II)	−0.000 (0.000)	0.000 (0.000)	0.000 (0.001)
Interaction (I × III)	−0.001 *** (0.000)	0.000 (0.000)	−0.001 *** (0.000)
Interaction (I × II × III)	5.39 × 10^−6^ * (2.88 × 10^−6^)	8.69 × 10^−7^ (2.83 × 10^−6^)	−0.000 ** (0.000)
Constant	56.097 *** (4.275)	52.203 *** (5.232)	38.328 *** (2.991)
Observations	6632	7680	2792
Countries	40	40	49

Note: Given that the health capacity is measured by year, while the remaining variables use the measurement of date, we keep its value without lagging. Standard errors are in parentheses, *** *p* < 0.01, ** *p* < 0.05, * *p* < 0.1.

**Table 6:** Descriptive analysis of the policy stringency over time.

Months	N	Mean	Standard Deviation	Minimum	Maximum
2021-06	2784	52.188	17.509	2.78	92.59
2021-07	5394	50.535	17.891	0	97.22
2021-08	5394	50.861	17.595	2.78	97.22
2021-09	5220	49.616	18.119	2.78	96.3
2021-10	5394	47.264	17.278	2.78	90.74
2021-11	5220	45.482	17.347	8.33	90.74
2021-12	5394	46.104	16.762	6.48	93.52
2022-01	5425	47.678	16.875	2.78	90.74
2022-02	4893	46.415	17.868	2.78	96.3
2022-03	5309	40.489	18.461	2.78	87.5
2022-04	1799	36.086	17.944	2.78	85.19

**Table 7:** Variance in policy responses over time (June 2021–April 2022) (Model 6).

Time	New_Deaths (−2) (I)	Vacci_Fpc (−2) (II)	Helt_Cp (III)	Interaction (I × II)	Interaction (I × III)	Interaction (I × II × III)
2021-06	0.056 *** (0.010)	−0.141 *** (0.030)	−0.070 *** (0.025)	0.000 (0.001)	−0.001 *** (0.000)	0.000 (0.000)
2021-07	0.068 *** (0.008)	−0.169 *** (0.017)	−0.075 *** (0.016)	0.002 ** (0.001)	−0.002 *** (0.000)	−3.36 × 10^−6^ (0.000)
2021-08	0.048 *** (0.007)	−0.102 *** (0.015)	−0.080 *** (0.017)	0.001 (0.000)	−0.001 *** (0.000)	−1.64 × 10^−6^ (5.30 × 10^−6^)
2021-09	0.075 *** (0.011)	−0.057 *** (0.017)	−0.032 * (0.019)	−0.000 (0.000)	−0.001 *** (0.000)	5.08 × 10^−6^ (4.58 × 10^−6^)
2021-10	0.021 (0.017)	0.018 (0.017)	0.003 (0.019)	0.001 ** (0.001)	−0.000 (0.000)	−0.000 ** (7.63 × 10^−6^)
2021-11	0.018 (0.016)	0.039 ** (0.016)	0.133 *** (0.019)	0.000 (0.000)	−0.000 (0.000)	7.67 × 10^−7^ (7.28 × 10^−6^)
2021-12	−0.007 (0.021)	−0.021 (0.015)	0.165 *** (0.017)	0.001 (0.000)	−0.000 (0.000)	−2.11 × 10^−6^ (7.17 × 10^−6^)
2022-01	0.056 * (0.031)	−0.019 (0.015)	0.154 *** (0.017)	0.001 (0.001)	−0.002 *** (0.001)	4.34 × 10^−6^ (8.99 × 10^−6^)
2022-02	0.097 *** (0.033)	−0.068 *** (0.019)	0.084 *** (0.020)	−0.000 (0.001)	−0.002 *** (0.001)	0.000 * (0.000)
2022-03	−0.085 *** (0.021)	−0.032 (0.022)	−0.053 ** (0.024)	0.002 *** (0.000)	0.002 *** (0.000)	−8.03 × 10^−6^ (6.73 × 10^−6^)
2022-04	−0.855 *** (0.313)	0.111 *** (0.039)	−0.152 *** (0.043)	0.013 *** (0.004)	0.012 *** (0.004)	−0.000 *** (0.000)

Note: We test Model 6 using the data grouped by date, and taking the policy responses proxied by stringency. The value of health capacity is employed without being lagged. For detailed information, see Tables S11–S15 in the Supplementary Materials. Standard errors are in parentheses, *** *p* < 0.01, ** *p* < 0.05, * *p* < 0.1.

## Data Availability

The data sources have been listed in the Table S2 of the Supplementary Materials, the datasets of which are available from the corresponding author upon reasonable request.

## Supplementary Materials

The following supporting information can be downloaded at: https://www.mdpi.com/article/10.3390/ijerph20054252/s1, Figure S1: Roots of Companion Matrix of (a) Model 1, (b) Model 2, (c) Model 3 and (d) Model 4; Figure S2: Graph of Orthogonalized Impulse-response in Model 1; Figure S3. Graph of Orthogonalized Impulse-response in Model 2; Figure S4. Graph of Orthogonalized Impulse-response in Model 3; Figure S5. Graph of Orthogonalized Impulse-response in Model 4; Figure S6. Roots of the Companion Matrix in Robust Model 1; Table S1. Countries and Territories in the Sample (n = 176); Table S2. Descriptions and explanations of variables; Table S3. Frequency of Categories of Stringency Index Components; Table S4. Correlation of Variables; Table S5. Estimated Models and Equations; Table S6. Panel unit root test using IPS and Fisher Methods (Model 1, Model 2, Model 3 and Model 4); Table S7. Panel VAR Lag Order Selection on Estimation Sample of Model 1, Model 2, Model 3 and Model 4; Table S8. Eigenvalue Stability Condition of Model 1, Model 2, Model 3 and Model 4; Table S9. Grange Causality Test of Model 1, Model 2 and Model 3; Table S10. Grange Causality Test of Model 4; Table S11. Variance in policy responses in June 2021, July 2021 and August 2021; Table S12. Variance in policy responses in September 2021 and October 2021; Table S13. Variance in policy responses in November 2021 and December 2021; Table S14. Variance in policy responses in January 2022 and February 2022; Table S15. Variance in policy responses in March 2022 and April 2022; Table S16. Panel unit root test using IPS and Fisher Methods (Robust Model 1); Table S17. Eigenvalue Stability Condition of Robust Model 1; Table S18. PVAR results of Robust Model 1; Table S19. Grange Causality Test of Robust Model 1; Table S20. Descriptive Statistics of Using Alternative Independent Variables; Table S21. Correlation of Alternative Independent Variables; Table S22. Results of Multinomial Logistic Regression on School closures (C1); Table S23. Results of Multinomial Logistic Regression on Workplace Closures (C2); Table S24. Results of Multinomial Logistic Regression on Cancellation of Public Events(C3); Table S25. Results of Multinomial Logistic Regression on Restrictions on Public Gatherings Size(C4); Table S26. Results of Multinomial Logistic Regression on Closures of Public Transport(C5); Table S27. Results of Multinomial Logistic Regression on Stay-at-home Requirement(C6); Table S28. Results of Multinomial Logistic Regression on Restrictions on Internal Movements(C7); Table S29. Results of Multinomial Logistic Regression on International Travel Controls(C8); Table S30. Results of Multinomial Logistic Regression on Public Information Campaigns(H1); Table S31. Variance in policy responses across regions (North America and South America); Table S32. PVAR results in Asia, Europe and Africa.

## Abbreviations

PVAR, panel data vector autoregression; NPIs, Non-pharmaceutical interventions; GHS, Global Health Security; OxCGRT, the Oxford Coronavirus Government Response Tracker; IRF, Impulse-Response Function.
