# Supplementary material for: Variation in Global Policy Responses to COVID-19: A Bidirectional Analysis
Source: Int J Environ Res Public Health. 2023 Feb 27;20(5):4252. doi: 10.3390/ijerph20054252 (PMC10001811; doi:10.3390/ijerph20054252)
Supplement: Supplementary file 1 [file ijerph-20-04252-s001.zip › ijerph-2166611-supplementary.pdf]

Supplementary Material

Table S1. Countries and Territories in the Sample (n=176).

Label	Name of Countries/Territories
AB	Afghanistan Albania Algeria Andorra Angola Argentina Aruba Australia Austria Azerbaijan Bahamas Bahrain Bangladesh Barbados Belarus Belgium Belize Benin Bermuda Bhutan Bolivia Bosnia and Herzegovina Botswana Brazil Brunei Bulgaria Burkina Faso Burundi
CD	Cambodia Cameroon Canada Cape Verde Central African Republic Chad Chile China Colombia Comoros Congo Costa Rica Cote d'Ivoire Croatia Cuba Cyprus Democratic Republic of Congo Denmark Djibouti Dominica Dominican Republic
EF	Ecuador Egypt El Salvador Eritrea Estonia Eswatini Ethiopia Faeroe Islands Fiji Finland France
GH	Gabon Gambia Georgia Germany Ghana Greece Greenland Grenada Guatemala Guinea Guyana Haiti Honduras Hungary
IJ	Iceland India Indonesia Iran Iraq Ireland Israel Italy Jamaica Japan Jordan
KL	Kazakhstan Kenya Kiribati Kosovo Kuwait Laos Latvia Lebanon Lesotho Liberia Libya Liechtenstein Lithuania Luxembourg
MN	Madagascar Malawi Malaysia Mali Malta Mauritania Mauritius Mexico Moldova Monaco Mongolia Morocco Mozambique Myanmar Namibia Nepal Netherlands New Zealand Nicaragua Niger Nigeria Norway
OP	Oman Pakistan Palestine Panama Papua New Guinea Paraguay Peru Philippines Poland Portugal
QR	Qatar Romania Russia Rwanda
ST	San Marino Saudi Arabia Senegal Serbia Seychelles Sierra Leone Singapore Slovenia Solomon Islands Somalia Somalia South Africa South Korea South Sudan Spain Sri Lanka Sudan Suriname Sweden Switzerland Syria Tajikistan Tanzania Thailand Togo Tonga Trinidad and Tobago Tunisia Turkey
UV	Uganda Ukraine United Arab Emirates United Kingdom United States Uruguay Uzbekistan Vanuatu Venezuela Vietnam
YZ	Yemen Zambia Zimbabwe

Table S2. Descriptions and explanations of variables.

Variable (Name)	Definition/Description	Source
stringency (Stringency Index)	the level of government non-pharmaceutical interventions with the score of 0 to 100.	OxCGRT
new_deaths (New Deaths)	The number of COVID-19 deaths of a country or territory on the observed day.	COVID-19 Data Repository (CSSE)
vacci_fpc (Fully Vaccinated Percentage)	The account of people who received all doses prescribed by the initial vaccination protocol in the total population of the country.	Self-computation on basis of Mathieu et al. 2021 and World bank 2022
helt_cp (Health Capacity)	The health capacity of clinics, hospitals and community care centers in coping with pandemics with the score of 0 to 100.	Global Health Security (GHS) Index 2021
new_cases (New Cases)	The number of confirmed cases of a country or territory on the observed day.	COVID-19 Data Repository (CSSE)
vacci_pc (Vaccinated Percentage)	The account of people who received at least one vaccine dose in the total population of the country.	Self-computation on basis of Mathieu et al. 2021 and World bank 2022
hosp (Hospital Bed)	Hospital beds per 10 000 population	WHO
C1 (School closing)	Record closings of schools and universities with the ordinal scale of 0 to 3.	OxCGRT
C2 (Workplace closing)	Record closings of workplaces with the ordinal scale of 0 to 3.	OxCGRT
C3 (Cancel public events)	Record cancelling public events with the ordinal scale of 0 to 2.	OxCGRT
C4 (Restrictions on gathering size)	Record limits on gatherings with the ordinal scale of 0 to 4.	OxCGRT
C5 (Close public transport)	Record closing of public transport with the ordinal scale of 0 to 2.	OxCGRT
C6 (Stay at home requirements)	Record orders to "shelter-in-place" and otherwise confine to the home with the ordinal scale of 0 to 3.	OxCGRT
C7 (Restrictions on internal Movement)	Record restrictions on internal movement between cities/regions with the ordinal scale of 0 to 2.	OxCGRT
C8 (Restrictions on international travel)	Record restrictions on international travel with the ordinal scale of 0 to 4.	OxCGRT
H1 (Public information campaign)	Record presence of public info campaigns with the ordinal scale of 0 to 2.	OxCGRT

Notes: The stringency index is a composite measure of nine of the response metrics, including school closures (C1); workplace closures (C2); cancellation of public events(C3); restrictions on public gatherings(C4); closures of public transport(C5); stay-at-home requirement(C6); public information campaigns(H1); restrictions on internal movements(C7); and international travel controls(C8). The variable of health capacity is defined within the field of clinics, hospitals and community care centers, this index contains available human resources for the broader healthcare system and facilities capacity. To compute the index, doctors per 100,000 people, nurses and midwives per 100,000 people and hospital beds per 100,000 people have been used, additionally, three questions are assessed by Economist Impact analyst in a qualitative approach according to official national sources.

Table S3. Frequency of Categories of Stringency Index Components.

Components of Stringency Index	Frequency of Individual Category (Percent)					Observations
	0	1	2	3	4	
C1	28.52	41.61	18.40	11.46		52,721
C2	17.34	38.48	35.34	7.83		52,274
C3	16.25	36.86	46.90			52,278
C4	20.01	4.24	11.57	33.47	30.71	52,249
C5	59.48	30.90	9.63			52,250
C6	46.54	24.22	27.90	1.35		52,227
C7	60.98	13.01	26.01			52,249
C8	1.41	31.50	32.06	25.12	9.92	52,259
H1	1.65	5.99	92.36			52,230

Table S4. Correlation of Variables.

	Stringency Index	New Deaths	Fully Vaccinated Percentage	Health Capacity
Stringency Index	1.000<br>(52226)			
New Deaths	0.140 ***<br>(50869)	1.000<br>(52316)		
Fully Vaccinated Percentage	−0.146 ***<br>(23949)	0.032 ***<br>(24136)	1.000<br>(24303)	
Health Capacity	0.116 ***<br>(50441)	0.226 ***<br>(50987)	0.357 ***<br>(23885)	1.000<br>(51850)

Note: star(0.01) obs pwcorr(correlation); Robust standard errors in parentheses; *** $p < 0.01$; ** $p < 0.05$; * $p < 0.1$.

Table S5. Estimated Models and Equations.

Models	Equations
Model 1	$S_{it} = \beta_0 + \beta_1 ND^{-1} + f_i + d_{ct} + \varepsilon_t$ (M1.1)<br>$ND_{it} = \beta_0 + \beta_2 S^{-1} + f_i + d_{ct} + \varepsilon_t$ (M1.2)
Model 2	$S_{it} = \beta_0 + \beta_3 ND^{-1} + \beta_4 ND^{-2} + \beta_5 VF^{-1} + \beta_6 VF^{-2} + f_i + d_{ct} + \varepsilon_t$ (M2.1)<br>$ND_{it} = \beta_0 + \beta_7 S^{-1} + \beta_8 S^{-2} + \beta_9 VF^{-1} + \beta_{10} VF^{-2} + f_i + d_{ct} + \varepsilon_t$ (M2.2)<br>$VF_{it} = \beta_0 + \beta_{11} S^{-1} + \beta_{12} S^{-2} + \beta_{13} ND^{-1} + \beta_{14} ND^{-2} + f_i + d_{ct} + \varepsilon_t$ (M2.3)
Model 3	$S_{it} = \beta_0 + \beta_{15} ND^{-1} + \beta_{16} ND^{-2} + \beta_{17} ND^{-3} + \beta_{18} HC^{-1} + \beta_{19} HC^{-2} + \beta_{20} HC^{-3} + f_i + d_{ct} + \varepsilon_t$ (M3.1)<br>$ND_{it} = \beta_0 + \beta_{21} S^{-1} + \beta_{22} S^{-2} + \beta_{23} S^{-3} + \beta_{24} HC^{-1} + \beta_{25} HC^{-2} + \beta_{26} HC^{-3} + f_i + d_{ct} + \varepsilon_t$ (M3.2)<br>$HC_{it} = \beta_0 + \beta_{27} S^{-1} + \beta_{28} S^{-2} + \beta_{29} S^{-3} + \beta_{30} ND^{-1} + \beta_{31} ND^{-2} + \beta_{32} ND^{-3} + \varepsilon_t$ (M3.3)
Model 4	$S_{it} = \beta_0 + \beta_{33} ND^{-1} + \beta_{34} ND^{-2} + \beta_{35} VF^{-1} + \beta_{36} VF^{-2} + \beta_{37} HC^{-1} + \beta_{38} HC^{-2} + \varepsilon_t$ (M4.1)<br>$ND_{it} = \beta_0 + \beta_{39} S^{-1} + \beta_{40} S^{-2} + \beta_{41} VF^{-1} + \beta_{42} VF^{-2} + \beta_{43} HC^{-1} + \beta_{44} HC^{-2} + \varepsilon_t$ (M4.2)<br>$VF_{it} = \beta_0 + \beta_{45} S^{-1} + \beta_{46} S^{-2} + \beta_{47} ND^{-1} + \beta_{48} ND^{-2} + \beta_{49} HC^{-1} + \beta_{50} HC^{-2} + \varepsilon_t$ (M4.3)<br>$HC_{it} = \beta_0 + \beta_{51} S^{-1} + \beta_{52} S^{-2} + \beta_{53} ND^{-1} + \beta_{54} ND^{-2} + \beta_{55} HC^{-1} + \beta_{56} HC^{-2} + \varepsilon_t$ (M4.4)
Model 5	$S_{it} = \beta_0 + \beta_{57} ND^{-2} + \beta_{58} VF^{-2} + \beta_{59} HC + \beta_{60} ND^{-2} * VF^{-2} + \beta_{61} ND^{-2} * HC + \beta_{62} ND^{-2} * VF^{-2} * HC + \varepsilon_t$ (M5)
Model 6	$S_{it} = \beta_0 + \beta_{63} ND^{-2} + \beta_{64} VF^{-2} + \beta_{65} HC + \beta_{66} ND^{-2} * VF^{-2} + \beta_{67} ND^{-2} * HC + \beta_{68} ND^{-2} * VF^{-2} * HC + \varepsilon_t$ (M6)

Notes: S denotes stringency (Stringency Index), ND represents new_deaths (New Deaths), VF is the abbreviation of vacci_fpc (Fully Vaccinated Percentage) and HC signifies helt_cp (Health Capacity). As for the order of lags, Model1 has the order of one lag, Model 2 has the order of two lag, Model 3

has the order of three lag and Model 4 has the order of two lag. Model 5 is a random effect regression and Model 6 is a fixed effect regression, with ND and VF lagged by two days.

Table S6. Panel unit root test using IPS and Fisher Methods (Model 1, Model 2, Model 3 and Model 4).

Model	Testing Method	Stringency Index	New Deaths	Fully Vaccinated Percentage	Health Capacity
Model 1	IPS	-1.6×10^2 ***	-1.6×10^2 ***		
	ADF-Fisher (P)	2.07×10^4 ***	2.09×10^4 ***		
	ADF-Fisher (Z)	−136.628 ***	−138.004 ***		
	ADF-Fisher (L*)	−327.528 ***	−330.069 ***		
	ADF-Fisher (Pm)	576.418 ***	581.018 ***		
Model 2	IPS	-1.2×10^2 ***	-1.1×10^2***	-	
	ADF-Fisher (P)	1.66×10^4 ***	1.46×10^4 ***	643.141 ***	
	ADF-Fisher (Z)	−120.962 ***	−113.119 ***	−10.750 ***	
	ADF-Fisher (L*)	−262.685 ***	−230.638 ***	−10.870 ***	
	ADF-Fisher (Pm)	458.858 ***	400.729 ***	12.420 ***	
Model 3	IPS	-	−91.593 ***		−91.916 ***
	ADF-Fisher (P)	1.23×10^4 ***	1.17×10^4 ***		1.17×10^4 ***
	ADF-Fisher (Z)	−102.674 ***	−100.003 ***		−100.350 ***
	ADF-Fisher (L*)	−196.253 ***	−185.162 ***		−184.967 ***
	ADF-Fisher (Pm)	338.499 ***	318.272 ***		317.920 ***
Model 4	IPS	-1.2×10^2***	-1.1×10^2 ***	-	-1.1×10^2 ***
	ADF-Fisher (P)	1.66×10^4***	1.46×10^4 ***	643.141 ***	1.51×10^4 ***
	ADF-Fisher (Z)	−120.962***	−113.119 ***	−10.750 ***	−115.852 ***
	ADF-Fisher (L*)	−262.685***	−230.638 ***	−10.870 ***	−239.208 ***
	ADF-Fisher (Pm)	458.858***	400.729 ***	12.420 ***	416.269 ***

Notes: Robust standard errors in parentheses.*** $p < 0.01$, ** $p < 0.05$, * $p < 0.1$.

In the Model 1, xtunitroot ips stringency, lags(1) demean, ADF regressions： 1 lags, W-t-bar. in the ADF-fisher tests of stringency, xtunitroot fisher stringency, dfuller drift lags(1) demean, ADF-Fisher (P) represents Inverse chi-squared (610), ADF-Fisher (Z) is Inverse normal, ADF-Fisher (L*) represents Inverse logit t(1529) and ADF-Fisher (Pm) represents Modified inv.chi-squared.

In the Model 2, xtunitroot ips stringency, lags(2) demean, ADF regressions： 1 lags, W-t-bar. The absence of fully vaccinated percentage in the test of IPS lies in insufficient observations. in the ADF-fisher tests of stringency, xtunitroot fisher vacci_fpc, dfuller drift lags(2) demean, ADF-Fisher (P) represents Inverse chi-squared (326), ADF-Fisher (Z) is Inverse normal, ADF-Fisher (L*) represents Inverse logit t(819) and ADF-Fisher (Pm) represents Modified inv.chi-squared.

In the Model 3, xtunitroot ips stringency, lags(3) demean, ADF regressions： 1 lags, W-t-bar. The absence of Stringency Index in the test of IPS lies in insufficient number of time periods to compute W-t-bar. In the ADF-fisher tests of stringency, xtunitroot fisher vacci_fpc, dfuller drift lags(2) demean, ADF-Fisher (P) represents Inverse chi-squared (602), ADF-Fisher (Z) is Inverse normal, ADF-Fisher (L*) represents Inverse logit t(1509) and ADF-Fisher (Pm) represents Modified inv.chi-squared.

In the Model 4, xtunitroot ips stringency, lags(2) demean, ADF regressions： 1 lags, W-t-bar. The absence of fully vaccinated percentage in the test of IPS lies in insufficient observations. in the ADF-fisher tests of stringency, xtunitroot fisher helt_cp, dfuller drift lags(2) demean, ADF-Fisher (P) represents Inverse chi-squared (610), ADF-Fisher (Z) is Inverse normal, ADF-Fisher (L*) represents Inverse logit t(1529) and ADF-Fisher (Pm) represents Modified inv.chi-squared.

Table S7. Panel VAR Lag Order Selection on Estimation Sample of Model 1, Model 2, Model 3 and Model 4.

Model	lag	CD	J	J pvalue	MBIC	MAIC	MQIC
Model 1	1	0.997	31.176	0.002	−98.605	7.176	−25.967
	2	0.997	23.390	0.003	−63.131	7.390	−14.705

	3	0.997	21.194	0.003	−22.066	13.194	2.146
	4	0.997	.	.	.	.	.
Model 2	1	0.887	141.286	1.92 × 10^{-17}	−20.685	87.286	44.541
	2	0.907	83.553	2.03e × 10^{-10}	−24.428	47.553	19.056
	3	0.955	30.728	0.000	−23.262	12.728	−1.520
	4	0.882	.	.	.	.	.
Model 3	1	−0.042	5999.998	0.000	5715.879	5945.998	5872.906
	2	0.008	3277.351	0.000	3087.938	3241.351	3192.623
	3	0.029	1918.908	0.000	1824.202	1900.908	1876.544
	4	−0.014	.	.	.	.	.
Model 4	1	0.919	253.248	1.09 × 10^{-29}	−33.127	157.248	81.782
	2	0.961	128.269	1.78 × 10^{-13}	−62.648	64.269	13.958
	3	0.984	93.466	5.72 × 10^{-13}	−1.99	61.466	36.311
	4	0.980	.	.	.	.	.

Note: For Model 1, the number of observations is 49763 with 174 panels. According to the selection order criteria, the random sample is shown as 22450-22749; For Model 2, the number of observations is 403 with 139 panels. According to the selection order criteria, the random sample is shown as 7-175; For Model 3, the number of observations is 37158 with 304 panels. According to the selection order criteria, the random sample is shown as 6-175; For Model 4, the number of observations is 390 with 138 panels. According to the selection order criteria, the random sample is shown as 7-175.

Table S8. Eigenvalue Stability Condition of Model 1, Model 2, Model 3 and Model 4.

**Model**	**Eigenvalue**		**Modulus**
	**Real**	**Imaginary**	
Model 1	0.056	−0.052	0.077
	0.056	0.052	0.077
Model 2	0.618	−0.188	0.646
	0.618	0.188	0.646
	−0.089	−0.564	0.571
	−0.089	0.564	0.571
	−0.407	−0.116	0.423
	−0.407	0.116	0.423
Model 3	0.766	0	0.766
	0.121	0.560	0.572
	0.121	−0.560	0.572
	−0.397	−0.408	0.569
	−0.397	0.408	0.569
	0.559	0	0.559
	−0.460	0	0.460
	−0.159	0.426	0.455
	−0.159	−0.426	0.455
Model 4	0.614	0.391	0.727
	0.614	−0.391	0.727
	−0.546	0.214	0.586
	−0.546	−0.214	0.586
	−0.110	−0.571	0.581
	−0.110	0.571	0.581
	0.276	0	0.276
	−0.022	0	0.022

Note: All values in the table above have been approximated with three digits. All the eigenvalues lie inside the unit circle, which proves that PVAR satisfies stability condition.

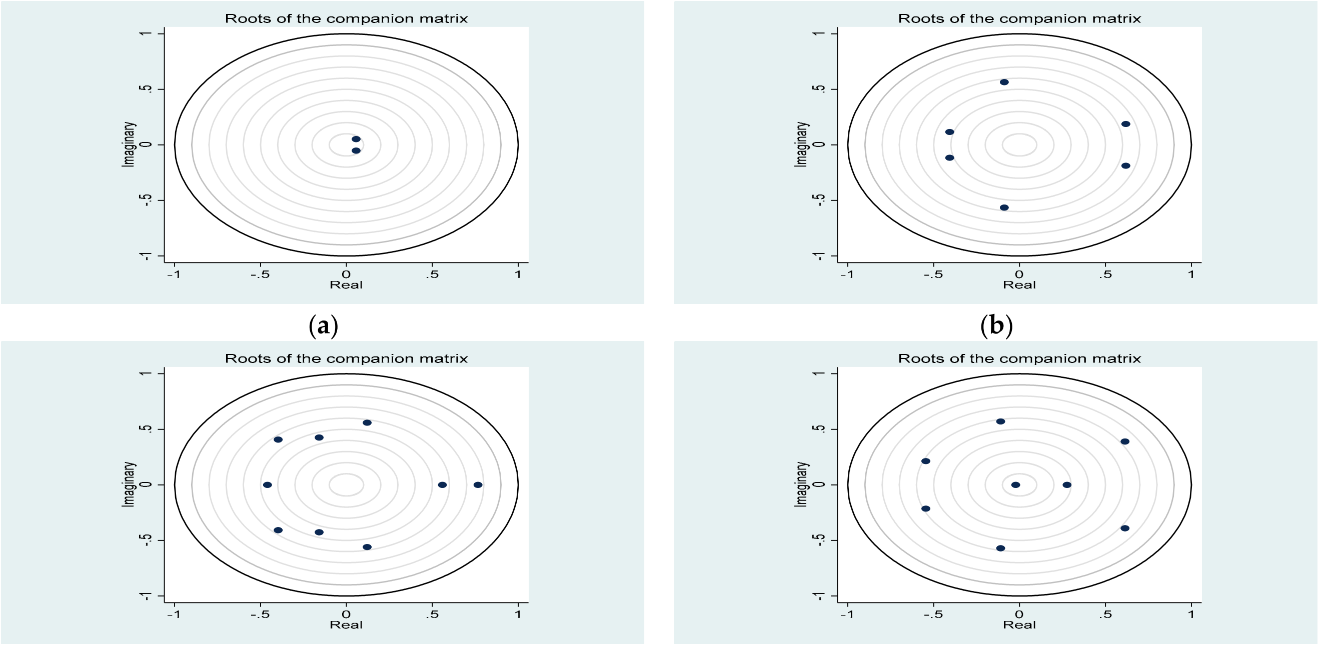

(c) (d)

Figure S1. Roots of Companion Matrix of (**a**) Model 1, (**b**) Model 2, (**c**) Model 3 and (**d**) Model 4.

Table S9. Grange Causality Test of Model 1, Model 2 and Model 3.

Model	Equation	Excluded variables	Chi2	df	Prob.
Model 1	stringency	stringency	n.a.	n.a.	n.a.
		new_deaths	22.754	1	0.000
		All	22.754	1	0.000
	new_deaths	new_deaths	n.a.	n.a.	n.a.
		stringency	473.344	1	0.000
		All	473.344	1	0.000
Model 2	stringency	stringency	n.a.	n.a.	n.a.
		new_deaths	17.417	2	0.000
		vacci_fpc	77.501	2	0.000
		All	114.942	4	0.000
	new_deaths	new_deaths	n.a.	n.a.	n.a.
		stringency	205.146	2	0.000
		vacci_fpc	134.810	2	0.000
		All	341.583	4	0.000
	vacci_fpc	vacci_fpc	n.a.	n.a.	n.a.
		stringency	807.070	2	0.000
		new_deaths	40.817	2	0.000
		All	846.670	4	0.000
Model 3	stringency	stringency	n.a.	n.a.	n.a.
		new_deaths	323.974	3	0.000
		helt_cp	1625.035	3	0.000
		All	2672.404	6	0.000
	new_deaths	new_deaths	n.a.	n.a.	n.a.
		stringency	116.946	3	0.000
		helt_cp	2341.553	3	0.000
		All	2480.523	6	0.000
	helt_cp	helt_cp	n.a.	n.a.	n.a.
		stringency	1545.326	3	0.000
		new_deaths	302.429	3	0.000
		All	2006.303	6	0.000

Table S10. Grange Causality Test of Model 4.

Equation	Excluded variables	Chi2	df	Prob.

Model 4	stringency	stringency	n.a.	n.a.	n.a.
		new_deaths	5.058	2	0.080
		vacci_fpc	111.496	2	0.000
		helt_cp	101.264	2	0.000
		All	264.992	6	0.000
	new_deaths	new_deaths	n.a.	n.a.	n.a.
		stringency	144.869	2	0.000
		vacci_fpc	13.488	2	0.001
		helt_cp	344.989	2	0.000
		All	568.516	6	0.000
	vacci_fpc	vacci_fpc	n.a.	n.a.	n.a.
		stringency	840.309	2	0.000
		new_deaths	55.946	2	0.000
		helt_cp	27.504	2	0.000
		All	939.842	6	0.000
	helt_cp	helt_cp	n.a.	n.a.	n.a.
		stringency	37.106	2	0.000
		new_deaths	172.788	2	0.000
		vacci_fpc	252.823	2	0.000
		All	340.946	6	0.000

Orthogonalized Impulse-response of PVAR Models

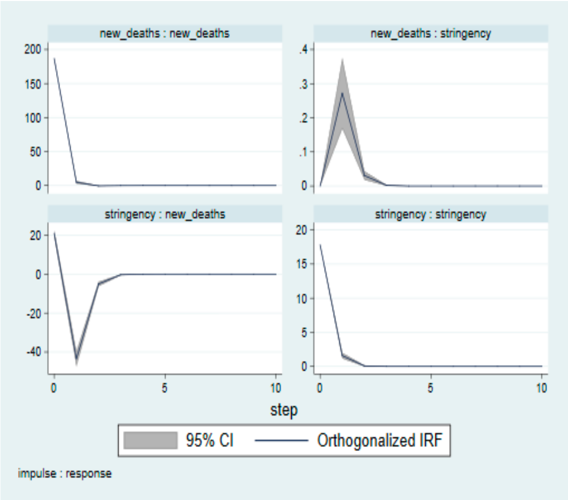

Figure S2. Graph of Orthogonalized Impulse-response in Model 1.

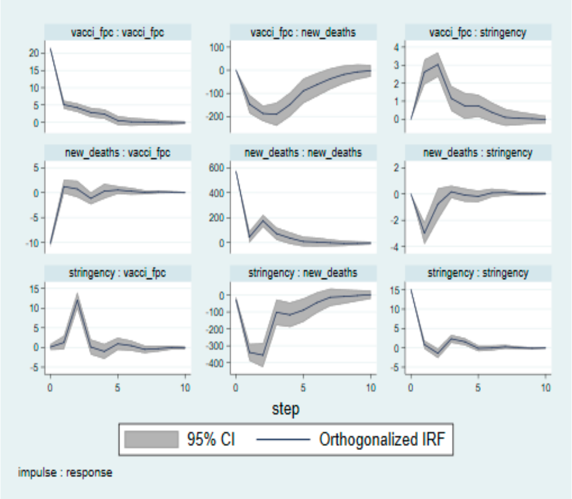

Figure S3. Graph of Orthogonalized Impulse-response in Model 2.

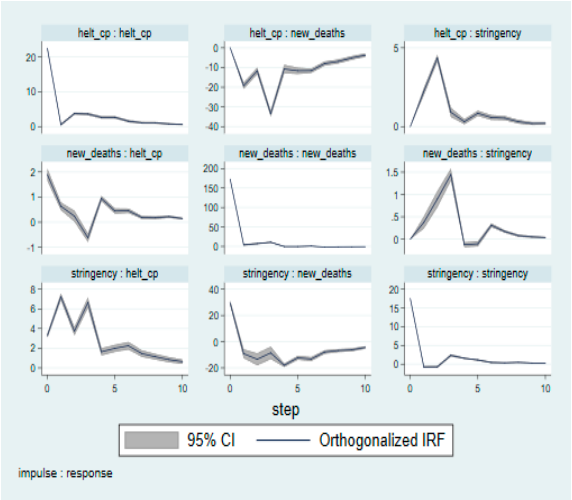

Figure S4. Graph of Orthogonalized Impulse-response in Model 3.

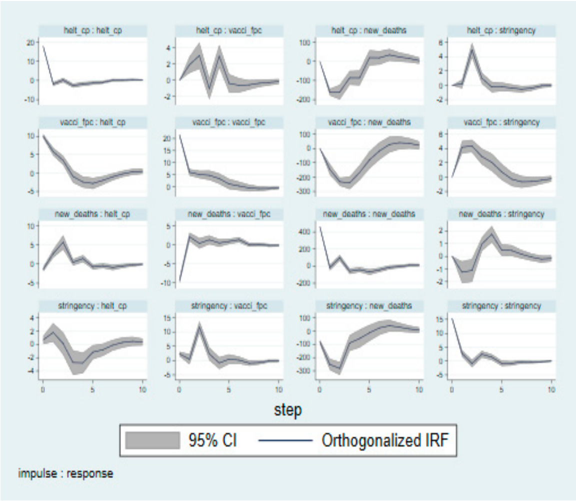

Figure S5. Graph of Orthogonalized Impulse-response in Model 4.

Note: The IRF confidence intervals are computed using 200 Monte Carlo draws from the distribution of the fitted reduced-form panel VAR model. The vertical axis is the intensity of impulse response and the horizontal axis signifies the lag period, while the solid line is the impulse response function curve, and the shaded areas represent 95% confidence intervals.

Detailed Results of Model 6

Table S11. Variance in policy responses in June 2021, July 2021 and August 2021.

**Independent variables**	**Dependent variable: stringency**		
	**June 2021**	**July 2021**	**August 2021**
new_deaths(−2) (I)	0.056 ***	0.068 ***	0.048 ***
	(0.010)	(0.008)	(0.007)
vacci_fpc (−2) (II)	−0.141 ***	−0.169 ***	−0.102 ***
	(0.030)	(0.017)	(0.015)
helt_cp (III)	−0.070 ***	−0.075 ***	−0.080 ***
	(0.025)	(0.016)	(0.017)
Interaction (I × II)	0.000	0.002 **	0.001
	(0.001)	(0.001)	(0.000)
Interaction (I × III)	−0.001 ***	−0.002 ***	−0.001 ***
	(0.000)	(0.000)	(0.000)
Interaction (I × II × III)	0.000	-3.36×10^{-6}	-1.64×10^{-6}
	(0.000)	(0.000)	(5.30×10^{-6})
Constant	61.164 ***	60.384 ***	59.523 ***

	(1.036)	(0.690)	(0.716)
Observations	1147	2543	2613
Date	14	31	31
r2 within	0.106	0.186	0.133
r2 between	0.276	0.010	0.092
r2 overall	0.107	0.185	0.131

Note: Standard errors are in parentheses, *** $p < 0.01$, ** $p < 0.05$, * $p < 0.1$.

Table S12. Variance in policy responses in September 2021 and October 2021.

Independent variables	Dependent variable: stringency	
	**September 2021**	**October 2021**
new_deaths(−2) (I)	0.075 ***<br>(0.011)	0.021<br>(0.017)
vacci_fpc (−2) (II)	−0.057 ***<br>(0.017)	0.018<br>(0.017)
helt_cp (III)	−0.032 *<br>(0.019)	0.003<br>(0.019)
Interaction (I × II)	−0.000<br>(0.000)	0.001**<br>(0.001)
Interaction (I × III)	−0.001 ***<br>(0.000)	−0.000<br>(0.000)
Interaction (I × II × III)	5.08×10^{-6}<br>(4.58×10^{-6})	−0.000 **<br>(7.63e × 10^{-6})
Constant	55.772 ***<br>(0.834)	48.378 ***<br>(0.846)
Observations	2475	2460
Date	30	31
r2 within	0.080	0.029
r2 between	0.231	0.048
r2 overall	0.081	0.029

Note: Standard errors are in parentheses, *** $p < 0.01$, ** $p < 0.05$, * $p < 0.1$.

Table S13. Variance in policy responses in November 2021 and December 2021.

Independent variables	Dependent variable: stringency	
	**November 2021**	**December 2021**
new_deaths(−2) (I)	0.018<br>(0.016)	−0.007<br>(0.021)
vacci_fpc (−2) (II)	0.039 **<br>(0.016)	−0.021<br>(0.015)
helt_cp (III)	0.133 ***<br>(0.019)	0.165***<br>(0.017)
Interaction (I × II)	0.000<br>(0.000)	0.001<br>(0.000)
Interaction (I × III)	−0.000<br>(0.000)	−0.000<br>(0.000)
Interaction (I × II × III)	7.67×10^{-7}<br>(7.28×10^{-6})	-2.11×10^{-6}<br>(7.17×10^{-6})
Constant	41.502 ***<br>(0.852)	44.738 ***<br>(0.833)
Observations	2392	2406
Date	30	31
r2 within	0.045	0.049
r2 between	0.306	0.150
r2 overall	0.046	0.050

Note: Standard errors are in parentheses, *** $p < 0.01$, ** $p < 0.05$, * $p < 0.1$.

Table S14. Variance in policy responses in January 2022 and February 2022.

Independent variables	Dependent variable: stringency	
	**January 2022**	**February 2022**
new_deaths(-2) (I)	0.056 *<br>(0.031)	0.097 ***<br>(0.033)
vacci_fpc (-2) (II)	−0.019<br>(0.015)	−0.068 ***<br>(0.019)

helt_cp (III)	0.154 ***<br>(0.017)	0.084 ***<br>(0.020)
Interaction (I × II)	0.001<br>(0.001)	−0.000<br>(0.001)
Interaction (I × III)	−0.002 ***<br>(0.001)	−0.002 ***<br>(0.001)
Interaction (I × II × III)	4.34×10^{-6}<br>(8.99×10^{-6})	0.000*<br>(0.000)
Constant	45.311 ***<br>(0.879)	47.600 ***<br>(1.087)
Observations	2369	2159
Date	31	28
r2 within	0.104	0.103
r2 between	0.043	0.111
r2 overall	0.102	0.103

Note: Standard errors are in parentheses, *** $p < 0.01$, ** $p < 0.05$, * $p < 0.1$.

Table S15. Variance in policy responses in March 2022 and April 2022.

Independent variables	Dependent variable: stringency<br>March 2022	April 2022
new_deaths(−2) (I)	−0.085 ***<br>(0.021)	−0.855 ***<br>(0.313)
vacci_fpc (−2) (II)	−0.032<br>(0.022)	0.111***<br>(0.039)
helt_cp (III)	−0.053 **<br>(0.024)	−0.152 ***<br>(0.043)
Interaction (I × II)	0.002 ***<br>(0.000)	0.013 ***<br>(0.004)
Interaction (I × III)	0.000<br>(0.000)	0.012 ***<br>(0.004)
Interaction (I × II × III)	-8.03×10^{-6}<br>(6.73×10^{-6})	−0.000 ***<br>(0.000)
Constant	43.918 ***<br>(1.287)	34.242<br>(2.501)
Observations	2102	649
Date	31	15
r2 within	0.050	0.080
r2 between	0.126	0.120
r2 overall	0.051	0.077

Note: Standard errors are in parentheses, *** $p < 0.01$, ** $p < 0.05$, * $p < 0.1$.

Robustness Check 1: Using Alternative Independent Variables

Table S16. Panel unit root test using IPS and Fisher Methods (Robust Model 1).

Model	Testing Method	Stringency Index	New Cases	Vaccinated Percentage	Hospital Bed
Robust Model 1	IPS	-1.6×10^2 ***	-1.7e $\times 10^2$ ***	Insufficient number of time periods to compute W-t-bar	Insufficient number of time periods to compute W-t-bar
	ADF-Fisher (P)	2.07×10^4 ***	2.20e $\times 10^4$ ***	2987.816 ***	-
	ADF-Fisher (Z)	−136.628 ***	−141.853 ***	−40.299 ***	-
	ADF-Fisher (L*)	−327.528 ***	−346.895 ***	−46.723 ***	-
	ADF-Fisher (Pm)	576.418 ***	611.528 ***	68.246 ***	-

Note: xtunitroot ips vacci_pc, lags(1) demean, xtunitroot fisher vacci_pc, dfuller drift lags(1) demean; Robust standard errors in parentheses.*** $p < 0.01$, ** $p < 0.05$, * $p < 0.1$

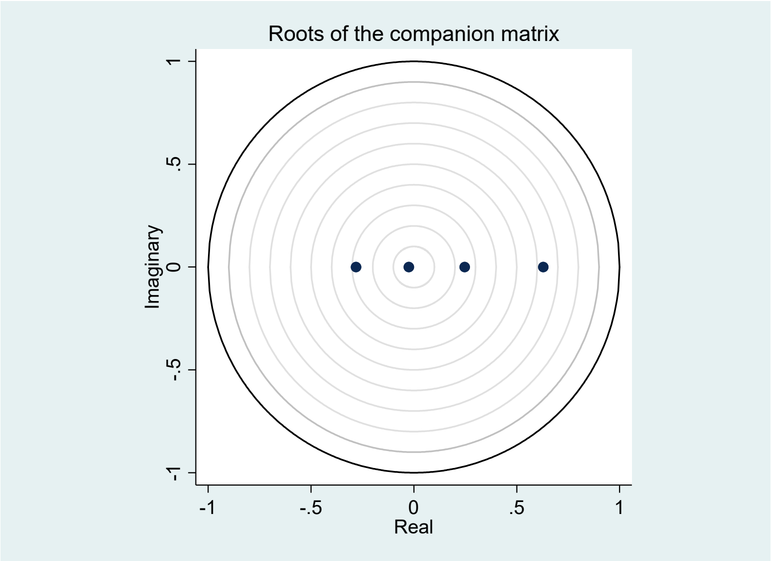

Figure S6. Roots of the Companion Matrix in Robust Model 1.

Table S17. Eigenvalue Stability Condition of Robust Model 1.

Model	Eigenvalue		Modulus
	Real	Imaginary	
Robust Model 1	0.629	0	0.629
	−0.281	0	0.281
	0.247	0	0.247
	−0.025	0	0.025

Table S18. PVAR results of Robust Model 1.

Model	Response to	Response of			
		stringency	new_cases	vacci_pc	hosp
Robust Model 1	stringency (−1)	0.320 ***	−3340.259 ***	−0.116 ***	0.008
		(0.036)	(181.190)	(0.041)	(0.005)
	new_cases (−1)	−0.000 ***	−0.001	−0.000 **	-1.25×10^{-7}
		(9.13×10^{-6})	(0.050)	(0.000)	(1.05×10^{-6})
	vacci_pc (−1)	0.202 ***	−2381.046 ***	0.226 ***	0.009 ***
		(0.028)	(135.505)	(0.029)	(0.003)
	hosp (−1)	1.073 ***	−18008.89 ***	−0.382	0.025
		(1.172)	(901.278)	(0.253)	(0.027)

Note: There are 4098 observations with 300 panels; Instrument: 1(1/2). (stringency new_cases vacci_pc hosp); Robust standard errors in parentheses; *** $p < 0.01$, ** $p < 0.05$, * $p < 0.1$.

Table S19. Grange Causality Test of Robust Model 1.

	Equation	Excluded variables	Chi2	df	Prob.
Robust Model 1	stringency	stringency	n.a.	n.a.	n.a.
		new_cases	21.051	1	0.000
		vacci_pc	53.057	1	0.000
		hosp	38.694	1	0.000
		All	63.781	3	0.000
	new_cases	new_cases	n.a.	n.a.	n.a.
		stringency	339.855	1	0.000
		vacci_pc	308.762	1	0.000
		hosp	399.260	1	0.000
		All	453.956	3	0.000
	vacci_pc	vacci_pc	n.a.	n.a.	n.a.
		stringency	7.861	1	0.005
		new_cases	6.009	1	0.014
		hosp	2.283	1	0.131

	All	23.398	3	0.000
hosp	hosp	n.a.	n.a.	n.a.
	stringency	2.602	1	0.107
	new_cases	0.014	1	0.905
	vacci_pc	7.719	1	0.005
	All	8.771	3	0.032

Robustness Check 2: Using the Dependent Variable's Components and Alternative Method

Table S20. Descriptive Statistics of Using Alternative Independent Variables.

Variable	Observation	Mean	Standard Deviation	Min	Max
stringency	52,226	47.063	17.973	0	97.22
new_cases	53,206	6009.327	29,123.200	0	1,383,886
vacci_pc	24,095	53.220	25.590	0.000	99.998
hosp	29,890	3.159	2.431	0.260	13.110

Table S21. Correlation of Alternative Independent Variables.

	Stringency Index	New Cases	Vaccinated Percentage	Hospital Bed
Stringency Index	1.000			
	(52,226)			
New Cases	0.099 ***	1.000		
	(51,756)	(53,206)		
Vaccinated Percentage	−0.071 ***	0.172 ***	1.000	
	(23,761)	(24,069)	(24,095)	
Hospital Bed	−0.108 ***	0.135 ***	0.161 ***	1.000
	(29,101)	(29,872)	(18,298)	(29,890)

Note: star(0.01) obs pwcorr(correlation); Robust standard errors in parentheses; *** $p < 0.01$, ** $p < 0.05$, * $p < 0.1$.

Table S22. Results of Multinomial Logistic Regression on School closures (C1).

Independent variables	Dependent variable: School closures (C1)		
	1	2	3
new_deaths	0.998 ***	1.002 ***	1.002 ***
	(0.000)	(0.000)	(0.000)
vacci_fpc	1.008 ***	1.004 ***	0.977 ***
	(0.001)	(0.001)	(0.001)
helt_cp	1.027 ***	1.014 ***	1.014 ***
	(0.001)	(0.001)	(0.002)
Intercept	0.638 ***	0.577 ***	0.649 ***
	(0.035)	(0.030)	(0.038)

Total N = 23,462; Notes: Robust standard errors in parentheses; *** $p < 0.01$, ** $p < 0.05$, * $p < 0.1$; Reference category for the model is countries and territories without taking response policy to Covid-19.

Table S23. Results of Multinomial Logistic Regression on Workplace Closures (C2).

Independent variables	Dependent variable: Workplace closures (C2)		
	1	2	3
new_deaths	1.005 ***	1.007 ***	1.007 ***
	(0.001)	(0.001)	(0.001)
vacci_fpc	1.002	0.999 ***	1.004 **
	(0.001)	(0.001)	(0.001)
helt_cp	1.002 ***	1.012 ***	1.003 *
	(0.001)	(0.001)	(0.002)
Intercept	3.064 ***	2.167 ***	0.483 ***
	(0.213)	(0.153)	(0.044)

Total N = 23,470; Notes: Robust standard errors in parentheses; *** $p < 0.01$, ** $p < 0.05$, * $p < 0.1$; Reference category for the model is countries and territories without taking response policy to COVID-19.

Table S24. Results of Multinomial Logistic Regression on Cancellation of Public Events(C3).

Independent variables.	Dependent variable: Cancellation of public events(C3)	
	1	2
new_deaths	1.007 ***<br>(0.001)	1.008 ***<br>(0.001)
vacci_fpc	0.992 ***<br>(0.001)	0.995 ***<br>(0.001)
helt_cp	1.006 ***<br>(0.001)	0.998 *<br>(0.001)
Intercept	3.538 ***<br>(0.274)	5.137 ***<br>(0.387)

Total N = 23,469; Notes: Robust standard errors in parentheses; *** $p < 0.01$, ** $p < 0.05$, * $p < 0.1$; Reference category for the model is countries and territories without taking response policy to COVID-19.

Table S25. Results of Multinomial Logistic Regression on Restrictions on Public Gatherings Size(C4).

Independent variables	Dependent variable: Restrictions on public gatherings size(C4)			
	1	2	3	4
new_deaths	0.998 ***<br>(0.000)	1.001 ***<br>(0.000)	1.000<br>(0.000)	1.001 ***<br>(0.000)
vacci_fpc	0.996 ***<br>(0.002)	0.983 ***<br>(0.001)	0.972 ***<br>(0.001)	0.984 ***<br>(0.001)
helt_cp	1.003 *<br>(0.002)	1.010 ***<br>(0.002)	1.013 ***<br>(0.001)	1.011 ***<br>(0.001)
Intercept	0.510 ***<br>(0.050)	1.380 ***<br>(0.101)	4.486 ***<br>(0.291)	3.419 ***<br>(0.219)

Total N = 23,447; Notes: Robust standard errors in parentheses; *** $p < 0.01$, ** $p < 0.05$, * $p < 0.1$; Reference category for the model is countries and territories without taking response policy to COVID-19.

Table S26. Results of Multinomial Logistic Regression on Closures of Public Transport(C5).

Independent variables	Dependent variable: Closures of public transport(C5)	
	1	2
new_deaths	1.002 ***<br>(0.000)	1.002 ***<br>(0.000)
vacci_fpc	0.986 ***<br>(0.001)	0.988 ***<br>(0.001)
helt_cp	0.999<br>(0.001)	0.992 ***<br>(0.001)
Intercept	0.916 **<br>(0.037)	0.452 ***<br>(0.023)

Total N = 23,468; Notes: Robust standard errors in parentheses; *** $p < 0.01$, ** $p < 0.05$, * $p < 0.1$; Reference category for the model is countries and territories without taking response policy to COVID-19.

Table S27. Results of Multinomial Logistic Regression on Stay-at-home Requirement(C6).

Independent variables	Dependent variable: Stay-at-home requirement(C6)		
	1	2	3
new_deaths	1.002 ***<br>(0.000)	1.002 ***<br>(0.000)	1.003 ***<br>(0.000)
vacci_fpc	0.997 ***<br>(0.001)	0.971 ***<br>(0.001)	0.957 ***<br>(0.003)
helt_cp	1.000<br>(0.001)	0.987 ***<br>(0.001)	0.999<br>(0.004)
Intercept	0.639 ***<br>(0.033)	2.810 ***<br>(0.131)	0.082 ***<br>(0.012)

Total N = 23,447; Notes: Robust standard errors in parentheses; *** $p < 0.01$, ** $p < 0.05$, * $p < 0.1$; Reference category for the model is countries and territories without taking response policy to COVID-19.

Table S28. Results of Multinomial Logistic Regression on Restrictions on Internal Movements(C7).

Independent variables	Dependent variable: Restrictions on internal movements(C7)	
	1	2
new_deaths	1.007 ***<br>(0.000)	1.006 ***<br>(0.000)
vacci_fpc	1.006 ***<br>(0.001)	0.991 ***<br>(0.001)
helt_cp	0.983 ***<br>(0.001)	0.990 ***<br>(0.001)
Intercept	0.270 ***<br>(0.015)	0.910 **<br>(0.036)

Total N = 23,467; Notes: Robust standard errors in parentheses; *** $p < 0.01$, ** $p < 0.05$, * $p < 0.1$; Reference category for the model is countries and territories without taking response policy to COVID-19.

Table S29. Results of Multinomial Logistic Regression on International Travel Controls(C8).

Independent variables	Dependent variable: International travel controls(C8)			
	1	2	3	4
new_deaths	1.004 ***<br>(0.001)	1.004 ***<br>(0.001)	1.005 ***<br>(0.001)	1.006 ***<br>(0.001)
vacci_fpc	0.957 ***<br>(0.002)	0.969 ***<br>(0.002)	0.970 ***<br>(0.002)	0.975 ***<br>(0.003)
helt_cp	0.967 ***<br>(0.003)	0.969 ***<br>(0.003)	0.987 ***<br>(0.003)	0.980 ***<br>(0.003)
Intercept	556.22 ***<br>(136.657)	447.136 ***<br>(109.745)	169.958 ***<br>(41.760)	51.980 ***<br>(13.229)

Total N = 23,469; Notes: Robust standard errors in parentheses; *** $p < 0.01$, ** $p < 0.05$, * $p < 0.1$; Reference category for the model is countries and territories without taking response policy to COVID-19.

Table S30. Results of Multinomial Logistic Regression on Public Information Campaigns(H1).

Independent variables	Dependent variable: Public information campaigns (H1)	
	1	2
new_deaths	1.043<br>(0.051)	1.047<br>(0.051)
vacci_fpc	0.982 ***<br>(0.006)	1.001<br>(0.006)
helt_cp	1.017 ***<br>(0.006)	1.036 ***<br>(0.005)
Intercept	6.365 ***<br>(1.904)	49.451 ***<br>(14.293)

Total N = 22,446; Notes: Robust standard errors in parentheses; *** $p < 0.01$, ** $p < 0.05$, * $p < 0.1$; Reference category for the model is countries and territories without taking response policy to COVID-19.

Robustness Check 3: Comparing other regions and employing alternative method

Table S31. Variance in policy responses across regions (North America and South America).

Independent variables	Dependent variable: stringency	
	North America	South America
new_deaths(−2) (I)	−0.010<br>(0.023)	0.036 ***<br>(0.010)
vacci_fpc (−2) (II)	−0.434 ***<br>(0.014)	−0.301 ***<br>(0.014)
helt_cp (III)	0.153<br>(0.215)	0.324 **<br>(0.156)
Interaction (I × II)	0.002 ***<br>(0.000)	−0.001 *<br>(0.000)
Interaction (I × III)	0.000<br>(0.000)	−0.00 1***<br>(0.000)
Interaction (I × II × III)	−0.000 ***	0.000 ***

	(7.35×10^{-6})	(6.10×10^{-6})
Constant	62.178 ***	58.114 ***
	(8.028)	(5.341)
Observations	2834	2677
Countries	19	12
r2 within	0.290	0.213
r2 between	0.003	0.334
r2 overall	0.111	0.293

Note: Standard errors are in parentheses，*** $p < 0.01$，** $p < 0.05$，* $p < 0.1$; The results are examined from using Model 5.

Table S32. PVAR results in Asia, Europe and Africa.

Region	Coefficients	Dependent Variables			
		**stringency**	**new_deaths**	**vacci_fpc**	**helt_cp**
Asia	stringency (−1)	−1.245 ***	3.498	−0.636 ***	−1.354 ***
		(0.413)	(5.621)	(0.187)	(0.321)
	new_deaths (−1)	0.015 ***	0.130*	−0.005 **	−0.009 ***
		(0.004)	(0.078)	(0.002)	(0.003)
	vacci_fpc (−1)	−0.138	−29.709 ***	−1.184 ***	−1.147 ***
		(0.477)	(6.244)	（0.260）	(0.384)
	helt_cp (−1)	−1.994 ***	4.597	0.932 ***	−0.336
		(0.618)	(8.837)	(0.340)	(0.543)
Europe	stringency (−1)	1.393 ***	−35.650 ***	2.077 ***	0.674 ***
		(0.181)	(4.658)	(0.251)	(0.172)
	new_deaths (−1)	−0.000	−0.527 **	0.027 **	−0.016 ***
		(0.004)	(0.237)	(0.013)	(0.003)
	vacci_fpc (−1)	0.017	−27.266 ***	1.047 ***	−0.305 ***
		(0.089)	(2.240)	(0.131)	(0.071)
	helt_cp (−1)	0.372	-0.878	−0.692 *	0.512 **
		(0.345)	(6.638)	(0.355)	(0.246)
Africa	stringency (−1)	−1.509 ***	−0.777 ***	−0.667 ***	−0.775 ***
		(0.335)	(0.282)	(0.219)	(0.189)
	new_deaths (−1)	0.045 **	-0.015	0.054 ***	0.038 ***
		(0.018)	(0.017)	(0.012)	(0.010)
	vacci_fpc (−1)	0.460 **	0.585 ***	0.471 **	0.596 ***
		(0.229)	(0.179)	(0.197)	(0.138)
	helt_cp (−1)	−1.269 ***	−0.614 ***	−1.361 ***	−0.447 ***
		(0.299)	(0.203)	(0.189)	(0.154)

Notes: In the sub-samples, there are 498 observations with 233 panels (Asia), 519 observations with 242 panels (Europe), 167 observations with 110 panels (Africa). These results can not be formally referenced due to that the stability of condition fails to be satisfied with applying the pvar method, its function in this section mainly serves to prove the heterogeneity of government interventions across these regions. Instrument: 1(1/1). (stringency new_deaths vacci_fpc health_capacity). Standard errors are in parentheses，*** $p < 0.01$，** $p < 0.05$，* $p < 0.1$.
